# EYA-1 is required for genomic integrity independent of H2AX signalling in *Caenorhabditis elegans*

**DOI:** 10.1007/s11033-024-09933-4

**Published:** 2024-09-24

**Authors:** Hannah R. Tatnell, Stevan Novakovic, Peter R. Boag, Gregory M. Davis

**Affiliations:** 1 Institute of Innovation, Science and Sustainability, Federation University, Churchill, VIC Australia; 2 Department of Biochemistry and Molecular Biology, Development and Stem Cells Program, Monash Biomedicine Discovery Institute, Clayton, VIC Australia; 3 Department of Health Sciences and Biostatistics, Swinburne University of Technology, Hawthorn, Australia

**Keywords:** DNA damage repair, Chromosomes, Reproduction, Development, Germ cells

## Abstract

**Background:**

Resolving genomic insults is essential for the survival of any species. In the case of eukaryotes, several pathways comprise the DNA damage repair network, and many components have high evolutionary conservation. These pathways ensure that DNA damage is resolved which prevents disease associated mutations from occurring in a de novo manner. In this study, we investigated the role of the Eyes Absent (EYA) homologue in *Caenorhabditis elegans* and its role in DNA damage repair. Current understanding of mammalian EYA1 suggests that EYA1 is recruited in response to H2AX signalling to dsDNA breaks. *C. elegans* do not possess a H2AX homologue, although they do possess homologues of the core DNA damage repair proteins. Due to this, we aimed to determine if *eya-1* contributes to DNA damage repair independent of H2AX.

**Methods and results:**

We used a putative null mutant for *eya-1* in *C. elegans* and observed that absence of *eya-1* results in abnormal chromosome morphology in anaphase embryos, including chromosomal bridges, missegregated chromosomes, and embryos with abnormal nuclei. Additionally, inducing different types of genomic insults, we show that *eya-1* mutants are highly sensitive to induction of DNA damage, yet show little change to induced DNA replication stress and display a mortal germline resulting in sterility over successive generations.

**Conclusions:**

Collectively, this study suggests that the EYA family of proteins may have a greater involvement in maintaining genomic integrity than previously thought and unveils novel roles of EYA associated DNA damage repair.

**Supplementary Information:**

The online version contains supplementary material available at 10.1007/s11033-024-09933-4.

## Introduction

The eukaryotic genome is constantly under stress, which can lead to lesions in DNA in the form of single or double stranded breaks (DSBs) [1]. Unrepaired and unchecked DNA lesions can lead to multiple diseased states such as cancer and developmental defects. To resolve these constant insults, several pathways exist and form an extensive network of DNA damage repair mechanisms that maintain genomic integrity, or in the case of irreparable DSBs, can initiate apoptosis to prevent mutations from proliferating [2, 3]. The Eyes Absent family of proteins (EYA) are characterised by their role as transcriptional cofactors as tyrosine phosphatases and are multifunctional regarding their contribution to development [4–6]. Initially named due to the absence of eyes in *eya* mutants in *Drosophila*, the EYA family of proteins regulate several pathways that contribute to developmental processes [5, 7–9]. EYA proteins have been implemented in signalling pathways and various cellular processes and their breakdown has been associated with several diseased states [5]. Less understood is the role EYA proteins play regarding the maintenance of genomic integrity. However, studies have shown that EYA proteins are recruitment to the site of DNA DSBs by the histone variant H2AX, which functions as a scaffold for the recruitment of DNA damage repair proteins [10–12]. In apoptosis, EYA proteins also initiate apoptosis by interacting with the highly conserved homeobox SIX family proteins. Due to the multi-functional roles exhibited by EYA proteins, characterising their precise role is challenging.

One of the key models for exploring developmental processes is the roundworm, *Caenorhabditis elegans*, which has been a valuable model for deciphering highly conserved genomic protection mechanisms [13, 14]. In *C. elegans* EYA proteins are represented by one protein, EYA-1, which functions as a homologue for mammalian EYA1-4. Investigations into *C. elegans* EYA-1 have revealed its involvement in egg laying, apoptosis, and response to heat stress [15–17]. In mammals, EYA-1 is recruited to DSBs by H2AX which functions as a primary signal for DSB repair [12]. However, the *C. elegans* genome lacks a H2AX orthologue, despite possessing many of the core proteins that participate in DSB repair [13]. Due to this, any role of EYA-1 in DNA damage repair pathways in *C. elegans* is likely to be independent of H2AX signalling. In this study, we show that absence of *eya-1* results in chromosomal abnormalities in anaphase embryos and results in a mortal germline phenotype. In addition to this, we also show that *eya-1* mutants exhibit hypersensitivity to induction of DNA damage, but not to DNA replication stress. Given the absence of H2AX in *C. elegans*, our findings unveil potential novel contributions of EYA-1 and its role in maintaining genomic integrity.

## Results and discussion

### EYA-1 is highly conserved and required for normal reproduction

**Fig. 1:**
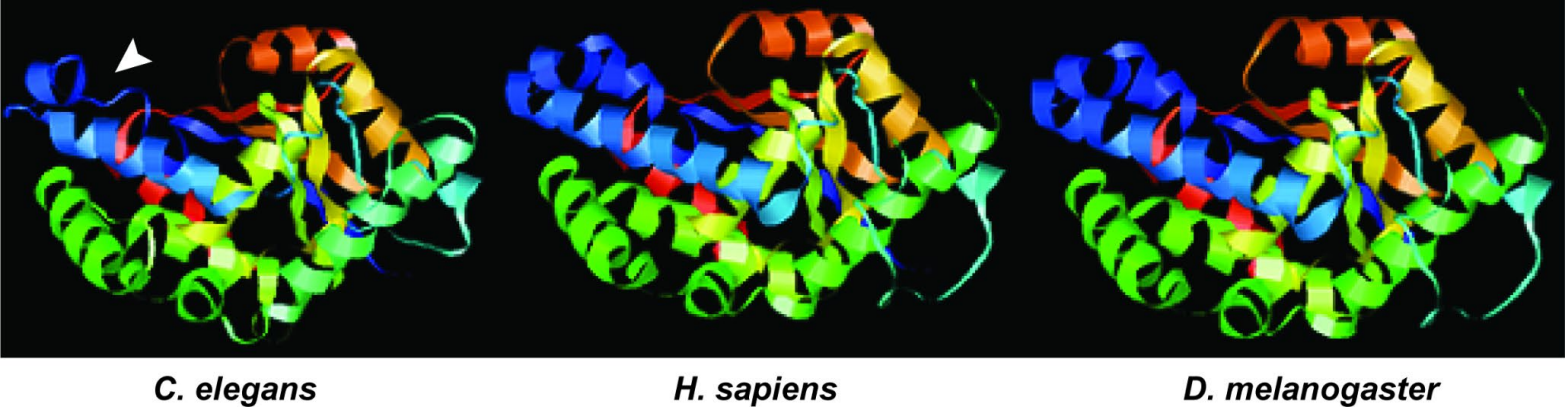
Homology modelling and active site conservation of EYA-1: Homology models showing structure of *C. elegans* EYA-1, *H. sapiens* EYA2, and *D. melanogaster* EYA-1. N-terminus is represented as blue and C-terminus is red. Arrow indicates variance of the shorter N-terminus alpha helix in *C. elegans* compared to *H. sapiens* and *D. melanogaster*

EYA family proteins possess an EYA domain (ED) which hosts a catalytic quintet of residues, which has previously been shown to exhibit high conservation of the EYA domain from plants, invertebrates and vertebrates [18]. The structure of EYA-1 has not been solved in either *C. elegans* or in higher eukaryotic species. To establish greater validity of EYA-1 as an ideal model for studying conserved features of EYA-1 homologues, structural models of *C. elegans* EYA-1 and homologues in *D. melanogaster* and *H. sapiens* were generated (Fig. 1). As expected, the structures of all three models exhibited similar overall features, with the notable difference of a smaller alpha-helix at the N-terminus in *C. elegans* compared to the N-terminus in *H. sapiens* and *D. melanogaster*. Despite this minor structural variation, the similarity suggests that *C. elegans* EYA-1 is a valid model for understanding roles conserved in higher eukaryotes.

**Fig. 2:**
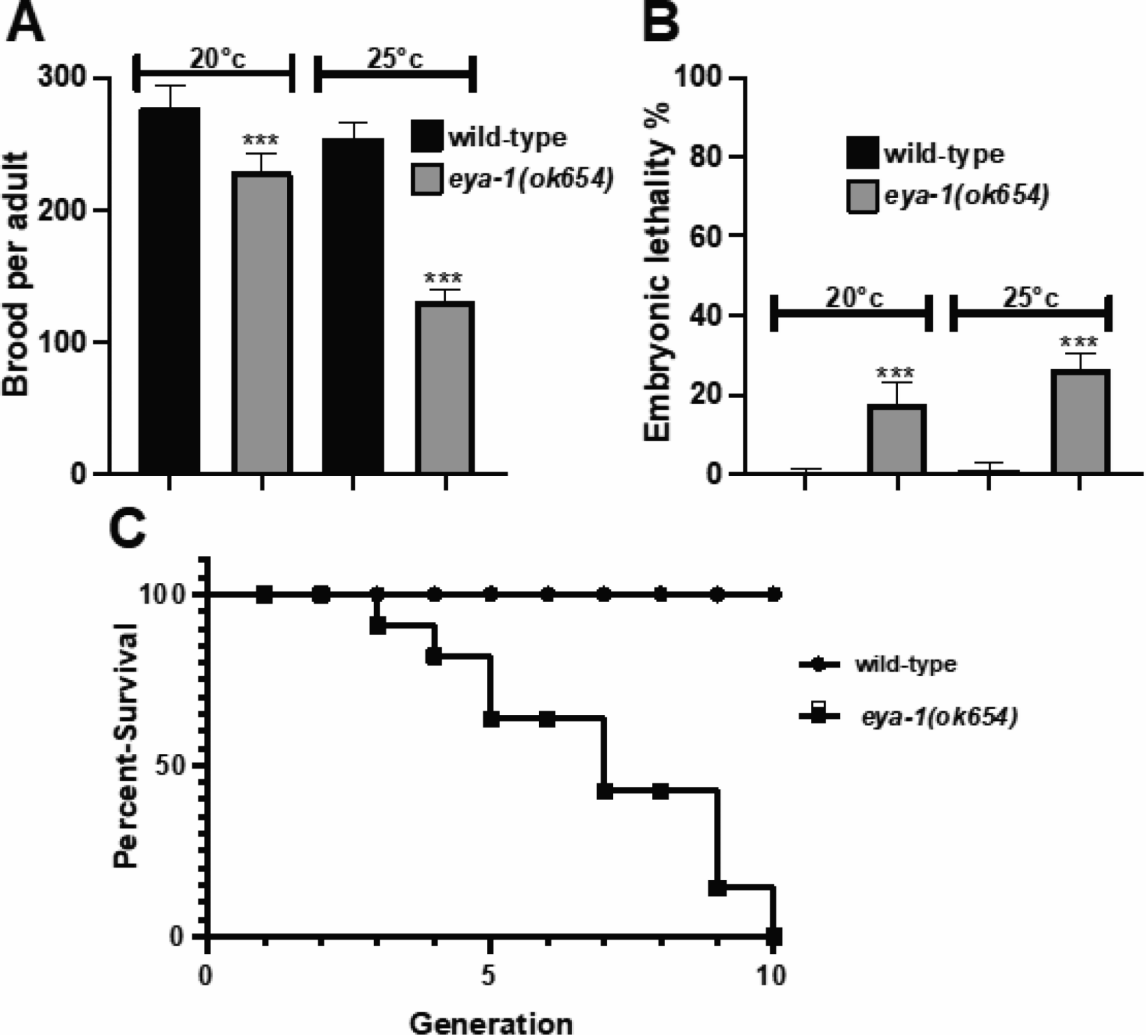
Reproductive capacity of *eya-1* deletion mutants and mortal germline: (**A**) Brood size of *eya-1(ok654)* mutants showing decreased brood which is exacerbated at higher temperatures. *** *p* = < 0.0001, error bars = SEM, *n* = 10. (**B**) Percentage of embryonic lethality from the brood of *eya-1(ok654)* mutants. *** *p* = < 0.0001, error bars = SEM, *n* = 10. (**C**) Mortal germline assay represented as percentage of survival over successive generations. *n* = 20

Having established structural conservation of EYA-1, we next assessed the requirement of *eya-1* for normal reproductive capacity. *C. elegans* hermaphrodites produce up to 300 progeny per animal with little genetic variability, making them an ideal model to assess the requirement of candidate proteins in reproductive tissue [14]. Therefore, we obtained an *eya-1* putative null mutant *eya-1(ok654)*, which has a large deletion of the EYA domain [15] and performed a brood size assay to determine if absence of *eya-1* impedes normal reproductive capacity (Fig. 2). This was conducted at 20 °C and a less permissive temperature of 25 °C, and interestingly, brood size was significantly decreased in *eya-1(ok654)* mutants compared to wild-type animals at both temperatures (Fig. 2A). Moreover, *eya-1(ok654)* mutants displayed elevated levels of embryonic lethality (Fig. 2B). Absence of EYA proteins in *Drosophila* are embryonic lethal showing that *eya-1(ok654)* mutants show a similar, albeit less severe trend [19], although our results do differ from those previously reported which suggest that *eya-1* mutants only display larval arrest [15]. Nonetheless, these findings suggest that *eya-1* is required for maintaining normal reproductive capacity in *C. elegans*.

### *eya-1* is required for embryonic chromosomal integrity and germline longevity

**Fig. 3:**
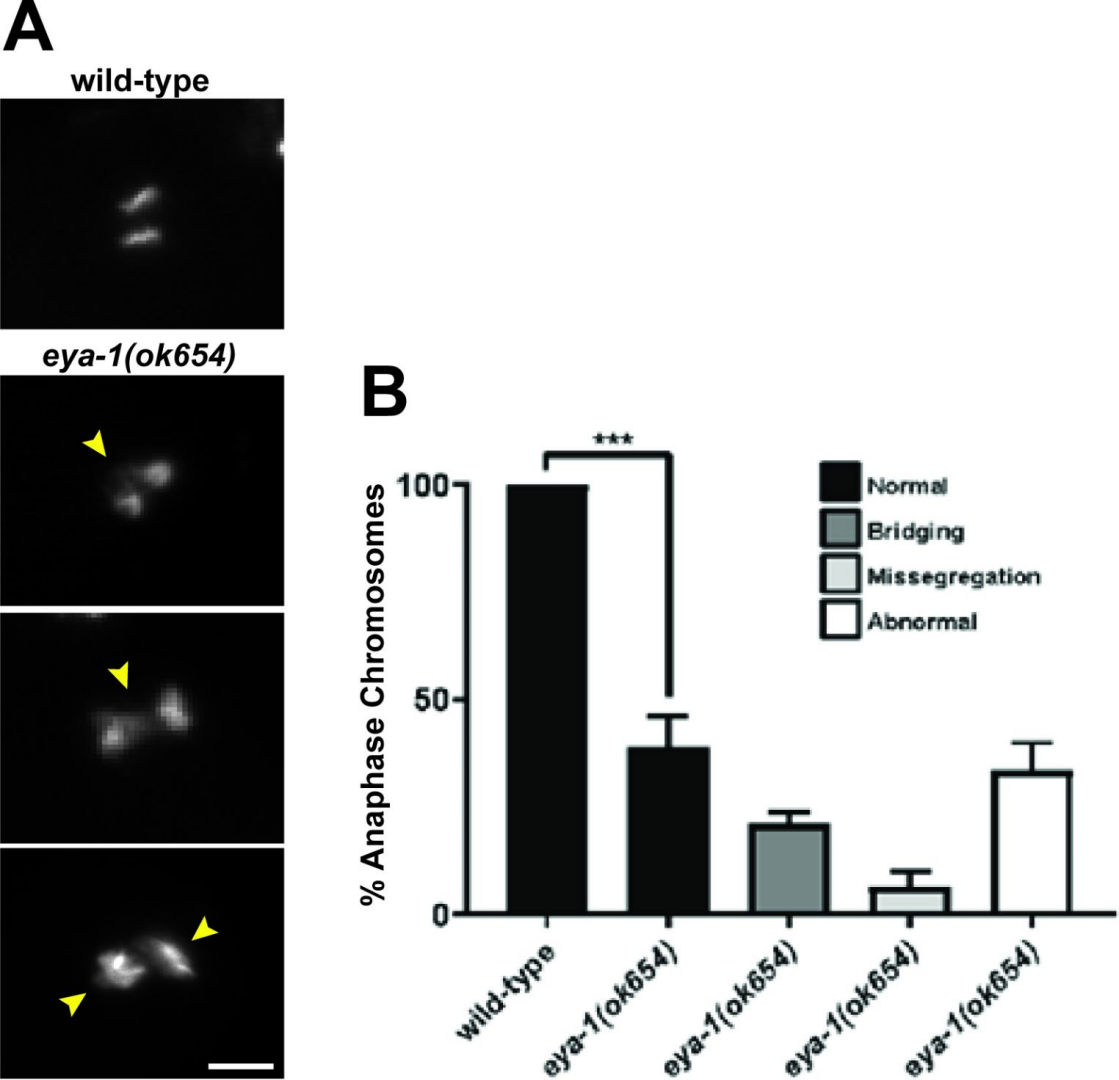
Abnormal chromosome morphology in *eya-1(ok654)* mutants: (**A**) Absence of *eya-1* results in abnormal chromosomes in anaphase embryos. Scale bar = 5 μm (**B**) Percentage of embryos scored and binned based on morphology. Arrows indicate chromosomal bridges, missegregated chromosomes, and abnormal nuclei (top to bottom) *n* = 200. *** *p* = < 0.0001, error bars = SEM

Abnormal reproductive capacity and embryonic lethality have been associated with chromosomal defects, which can be observed in anaphase stage embryos during meiosis II [20]. To investigate whether the germline and embryonic phenotypes observed in *eya-1(ok654)* mutants were due to any visible chromosome morphological defects, we examined embryos in the 1 to 2 cell stage of division. Strikingly, *eya-1(ok654)* mutants exhibit a broad range of adverse morphologies, including chromosomal bridges, embryos with missegregated chromosomes, and embryos with abnormal nuclei (Fig. 3A, B). We also assessed the germline nuclei of *eya-1(ok654)* mutants and interestingly found no abnormalities in pachytene nuclei or diakinetic oocytes (data not shown). However, we did observe a mild, low penetrance HIM phenotype (High Incidence of Males) at 25 °C which suggests that germline chromosomal segregation defects may be present (Fig. S1A). In addition to this, we also observed a mild increase in germ cell apoptosis when compared to wild-type animals (Fig. S1C). Nonetheless, we cannot determine if any unobservable defects are present in the preliminary stages of oocytes, or if these observed abnormal embryonic chromosomes are restricted to the anaphase stage events of meiosis II. Nonetheless, the reduced brood size of *eya-1(ok654)* mutants, coupled with an increase in germ cell apoptosis suggests that germline processes may be impeded in the absence of *eya-1*.

To investigate this further, *eya-1(ok654)* mutants were subjected to a mortal brood size assay (Fig. 2C), where individual *eya-1(ok654)* mutants were transferred from each generation alongside wild-type animals until sterility occurred. The rationale behind is based on the accumulation of inherited germline mutations throughout generations, ultimately render the strain sterile [21, 22]. We observed a marked reduction in brood size that decreased with each generation. This trend persisted until the 10th generation, at which point *eya-1(ok654)* mutants were sterile, with no recorded change in the brood of wild-type animals. This suggests that *eya-1(ok654)* mutants accumulate germline defects that are transferred to each generation. This is interesting as *eya-1* expression is typified from early embryogenesis and is most active during somatic development [15]. Unfortunately, we were unsuccessful in generating a functional EYA-1 germline antibody to investigate this further. However, it is possible that the absence of EYA-1 activity in the somatic gonad may have a generational accumulative influence of germline processes independent of *eya-1* germline expression. Despite this, the absence of *eya-1* in *C. elegans* results in sterility over successive generations, raising novel concerns regarding its influence of germ cell function and longevity.

### *eya-1(ok654)* mutants are hypersensitive to DNA damage

**Fig. 4:**
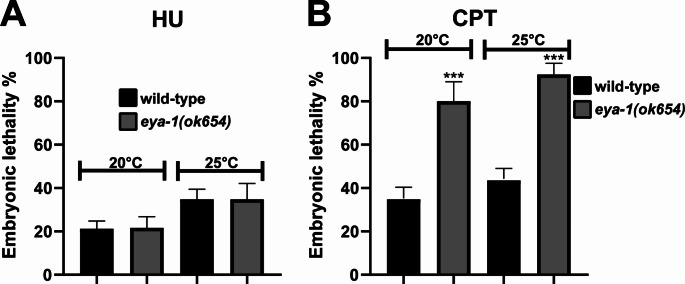
Hypersensitivity of DNA damage in *eya-1(ok654)* mutants: (**A**) DNA replication stress induced with hydroxyurea (HU) showing little change from wild-type animals. *n* = 20, *** *p* = < 0.0001, error bars = SEM. (**B**) Hypersensitivity of *eya-1(ok654)* mutants to DNA breaks induced with camptothecin (CPT) with elevated embryonic lethality in *eya-1(ok654)* mutants. *n* = 20, *** *p* = < 0.0001, error bars = SEM. Replicated in triplicates

The observation of embryonic lethality and abnormal anaphase chromosomes in *eya-1(ok654)* mutants suggests a role for *eya-1* in DNA damage repair. To determine if these genomic insults were due to replication stress or DNA damage, we aimed to induce both insults in *eya-1(ok654)* mutants and assess the severity of each insult. Therefore, *eya-1(ok654)* mutants were subjected to hydroxyurea (HU) to induce replication stress and camptothecin (CPT), which induces single stranded breaks and indirectly leads to double stranded breaks [23] (Fig. 4). This was performed at both 20 °C and 25 °C, consistent with our prior results, and both temperatures showed the same trend upon exposure to each agent. HU exposure led to a minor increase in unhatched embryos in *eya-1(ok654)* mutants similar to the trend observed in wild-type animals (Fig. 4A). However, exposure to CPT led to a more noticeable increase in unhatched embryos (Fig. 4B). This hypersensitivity to CPT, coupled with observed embryonic chromosomal defects suggests a strong requirement of EYA-1 in single or double strand DNA break repair. To ensure that this trend was specific to *eya-1* and not a background mutation we performed the same assay on wild-type animals that were subjected to RNAi of *eya-1* and observed a similar trend (Fig S1B). Current understanding of EYA-1 and its role in DNA repair involves H2AX as a substrate for EYA-1 as part of the primary dsDNA break signal [11, 24]. Since *C. elegans* lacks H2AX, this might infer that EYA-1 has novel roles in DNA break repair beyond H2AX signalling.

This correlation between EYA-1 and DNA damage repair may be more varied than first thought. Increased DNA damage in the absence of H2AX in *C. elegans* is a significant point of interest as it potentially adds a new role of EYA proteins in DNA repair. In mammals, after the occurrence of DSBs, EYA-1 recruits a series of proteins associated with DSB repair, and the stimulus for this association of EYA-1 is phosphorylation of H2AX. This allows EYA-1 to function as a scaffold for the core DSB machinery to assemble and facilitate DSB repair [5, 11, 24]. Although *C. elegans* do not possess H2AX, many of the proteins EYA-1 recruits in this process for DSB repair are conserved and therefore present in *C. elegans* [13]. Due to this, it is possible that EYA-1 may have a conserved role in DNA repair that goes beyond that of H2AX phosphorylation and may participate further upstream in DNA repair pathways.

EYA-1 is required for the initiation of apoptosis by its association with *ceh-*34 [17]. We therefore assessed germ cell apoptosis in *eya-1(ok654)* mutants and observed a mild increase in germ cells undergoing apoptosis (Fig. S1D, E). Due to this, we cannot rule out the possibility that this hypersensitivity to induced DNA damage in *eya-1* mutants might be due to deficient induction of apoptosis as normally initiated by *eya-1* and *ceh-34*. In this regard, it might well be that cells with DNA damage beyond repair that would otherwise undergo apoptosis might be unchecked and allowed to persist. Therefore, our observations may be due to an overaccumulation of DNA damage in cells that would otherwise be eradicated in wild-type animals. However, the specific sensitivity to induction of DNA damage in *eya-1* mutants coupled with the lack of sensitivity to induction of replication stress suggests a correlation between *eya-1* and single or double stranded DNA repair independent of H2AX signalling.

## Conclusion

EYA proteins are multi-functional and participate in numerous pathways. Due to this, deciphering the precise role that EYA-1 plays within the network of DSB repair will enhance understanding of both EYA-1 and the overall repair process. Nonetheless, this study shows that EYA-1 likely has a role in DNA repair beyond its previously characterised role in association with H2AX signalling. Therefore, we conclude that the role of EYA-1 in resolving DNA damage may be more complex than current mechanisms assume, and deciphering its various contributions to this pathway will enhance understanding of DNA repair processes.

## Materials and methods

### Strains

Strains used in this study were maintained 20 and 25 °C under standard conditions [25]. The Bristol N2 strain was used for the wild-type strain and the *eya-1(ok654)* deletion mutant was obtained from the CGC [26] and backcrossed five times prior to performing all experiments. Genotyping of *eya-1(ok654)* animals was performed using the following primers. Forward: GCACGGCAAATTACGAAAGC and Reverse: GCCGTGCTTAACAAACTCCA.

### Embryonic DNA analysis

Dissections were performed as described previously [27]. In short, 1-day old adult animals were isolated and anesthetized in 0.001% tetramisole in M9 buffer on 22 × 22 mm coverslips and dissected using two syringe needles. Coverslips were then placed onto microscopy slides precoated poly-L-lysine and snap frozen in liquid nitrogen then incubated in methanol for 20 min. Slides were washed twice with washing buffer (0.1% Tween-20 in PBS) for 10 min and covered with DAPI diluted in normal goat serum, then incubated at room temperature in a humidity chamber for 1 h. Slides were washed twice with wash buffer for 10 min and mounted on coverslips using Dako Fluorescent Mounting Media (Dako, Glostrup, Denmark). Slides were examined using a Zeiss Axiolab microscope with a pE-300 lite LED fluorescence adaptor (Zeiss, Oberkochen, Germany). Images were captured using a 1.4-MP (1,360 × 1,024) Tucsen 2/3 monochrome CCD camera (Tucson, Fujian, China). Anaphase embryos were binned as normal (two distinct sets of chromosomes), bridging (lagging strands of DNA between chromosomes) missegregation (chromosomal bodies not correctly separated), and abnormal (clumped or disordered yet did not clearly resemble the above descriptions). Analysis was conducted 3 times using the above protocol on wild-type and *eya-1(ok654)* animals with ∼ 100 L1 worms on NGM plates feeding on OP50 bacteria until reaching adulthood prior to dissection and DAPI staining. Counting of anaphase chromosomes occurred until *n* = 200 was achieved.

### Brood and embryonic lethality

Brood and embryonic lethality counts were performed at 20 and 25 °C. Synchronized populations of each strain were grown at 20 °C until the fourth larval stage then placed onto NGM plates pre-seeded with OP50. Each worm was then transferred to new plate every 12 h and scored for progeny after 48 h. This was repeated until each worm failed to lay any new progeny. Total progeny was recorded as viable progeny and unhatched embryos. *n* = 10, performed in three replicates.

### Induction of DNA damage

Inducing DNA damage was achieved by placing worms to plates prepared with 25 mM hydroxyurea (Sigma-Aldrich, Missouri, USA), or 50 µM camptothecin (Sigma-Aldrich, Missouri, USA) and repeated three times in triplicates as previously described [28]. In short, twenty L4 staged wild-type and *eya-1* mutants were placed on NGM plates enriched with each reagent, seeded with OP50 and incubated at 20 °C and 25 °C for 16 h. Worms were then transferred to seeded NGM plates for 4 h for recovery, then transferred again to fresh seeded NGM plates. Plates with embryos were then incubated at 20–25 °C accordingly for 24 h, then scored for embryonic lethality and hatching rates.

### *C. elegans* mortal germline assay

Mortal germline assays were conducted by placing 10 individual L1 wild-type and *eya-1* mutants on seeded NGM plates at 25 °C until they laid progeny. One representative worm from the progeny of each plate were transferred to new plates until they matured and started to lay progeny. The process was then repeated until *eya-1* mutants were sterile. Brood size and rates of embryonic lethality were recorded from the brood of each generation.

### RNA interference

RNAi was performed by the plate feeding method and bacterial clones were sourced from the ORFeome library [29]. RNAi clones were grown in 2 x TY media plus 100 mg/mL ampicillin overnight at 37 °C on NGM plates (3% bacto-agar, 86 mM NaCl, 42 mM Na2HPO4, 22 mM KH2PO4, and 1 mM MgSO4) containing 100 mg/mL ampicillin plus 4 mM IPTG. Wild-type animals were fed an empty vector and grown alongside animals treated with RNAi. Approximately, 100 synchronized L1 wild-type animals were then transferred to seeded plates in duplicates and grown at 20 °C and 25 °C prior to analysis and performed in three separate replicates.

### Acridine orange staining

Germ cells apoptosis used acridine orange as described previously [30]. In short, 20 day 1 adult worms were stained with 1 ml of 100µM acridine orange (Sigma-Aldrich, Missouri, USA) on NGM plates seeded with OP50 and for 1 h, then washed in M9 buffer and placed on 2% agarose gel pads in 0.03% tetramisole and scored using DIC and fluorescence microscopy. This was replicated for 20 °C and 25 °C in duplicates and performed in three separate replicates.

### Statistical analysis and software

Generation of graphs and statistical analysis utilised the Prism 5 software package (GraphPad, San Diego, CA, USA). Statistical significance was determined using a student t-test and errors were represented as standard error of the mean. Images were processed using Adobe Photoshop, and Adobe Illustrator was used to assemble figures (Adobe, San Jose, CA, USA). Multiple sequence alignment was achieved using Clustral Omega [31] and protein homology models were generated using Swiss-Model [32].

## Electronic supplementary material

Below is the link to the electronic supplementary material.

Supplementary Figure 1: Knockdown on *eya-1* phenocopies *eya-1(ok654)* mutants and *eya-1* mutants display enhanced germ cell apoptosis and a mild HIM phenotype: (**A**) *eya-1(ok654)* mutants show a low penetrance HIM phenotype compared to wild-type animals at 25°C. *him-14(RNAi)* represents positive control. *** *p* = < 0.0001, error bars = SEM, *n* = 700 replicated in triplicates. (**B**, **C**) Knockdown of *eya-1* phenocopies DNA damage sensitivity to HU and CPT as observed in Fig. 4. *n* = 20, *** *p* = < 0.0001, error bars = SEM. Replicated in triplicates. (**D**, **E**) Germ cell death assessed via acridine orange (AO) staining in *eya-1(ok654)* mutants. Scale bar = 20 μm, *n* = 60, error bars = SEM

## Data Availability

No datasets were generated or analysed during the current study.
