# Supplementary figures and images for: EYA-1 is required for genomic integrity independent of H2AX signalling in Caenorhabditis elegans
Source: Mol Biol Rep. 2024 Sep 24;51(1):1009. doi: 10.1007/s11033-024-09933-4 (PMC11422256; doi:10.1007/s11033-024-09933-4)

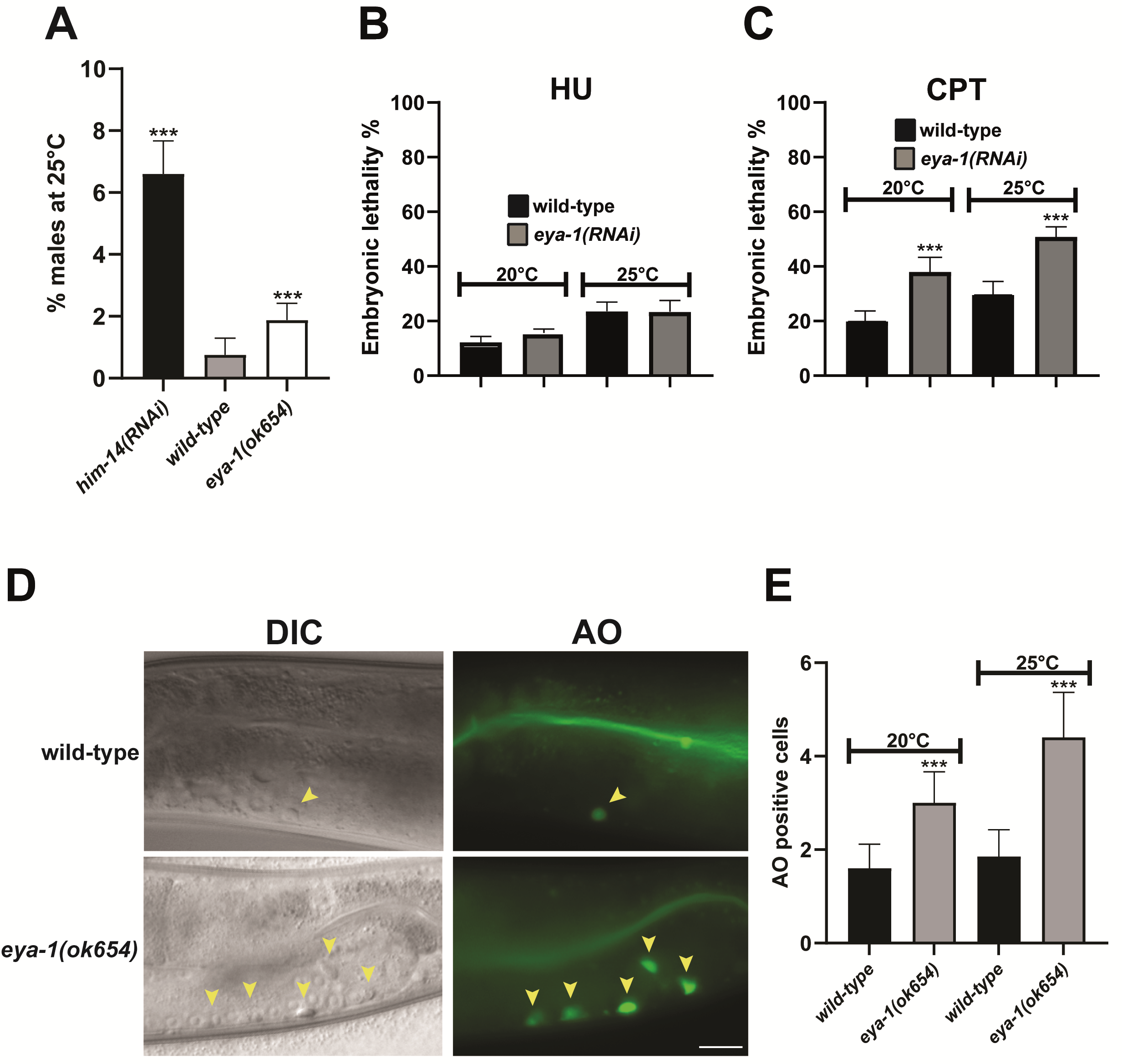

Supplement: Supplementary file 1 — Supplementary Figure 1: Knockdown on eya-1 phenocopies eya-1(ok654) mutants and eya-1 mutants display enhanced germ cell apoptosis and a mild HIM phenotype: (A) eya-1(ok654) mutants show a low penetrance HIM phenotype compared to wild-type animals at 25°C. him-14(RNAi) represents positive control. *** p = < 0.0001, error bars = SEM, n = 700 replicated in triplicates. (B, C) Knockdown of eya-1 phenocopies DNA damage sensitivity to HU and CPT as observed in Fig. 4. n = 20, *** p = < 0.0001, error bars = SEM. Replicated in triplicates. (D, E) Germ cell death assessed via acridine orange (AO) staining in eya-1(ok654) mutants. Scale bar = 20 μm, n = 60, error bars = SEM [file 11033_2024_9933_MOESM1_ESM.png]
